# Multi-scenario flexible contract coordination for determining the quantity of emergency medical suppliers in public health events

**DOI:** 10.3389/fpubh.2024.1334583

**Published:** 2024-03-20

**Authors:** Hanping Hou, Kun Zhang, Xuewei Zhang

**Affiliations:** School of Economics and Management, Beijing Jiaotong University, Beijing, China

**Keywords:** emergency medical suppliers, public health events, procurement of emergency medical supplies, optimal number of suppliers, supply risk, option contract

## Abstract

Determining the optimal number of emergency medical suppliers for the government to contract with in the context of public health events poses a challenging problem. Having too many suppliers can result in increased costs, while having too few suppliers can potentially expose the government to supply risks. Striking the right balance between these two factors is crucial in ensuring efficient and reliable emergency response and management. This study examines the process of determining the appropriate number of suppliers in emergency medical supply chain. By incorporating option contracts and employing the total cost of government procurement as the objective function, the analysis focuses on the impact of relevant parameters on the optimal number of suppliers. Furthermore, the study investigates the optimal supplier quantities under different types of option contracts. The proposed decision model for determining the optimal number of suppliers in this paper considers three key factors: the supply risk associated with emergency medical supplies, the reserve cost of government procurement, and the responsiveness of emergency medical supplies. Additionally, a method is introduced for selecting the quantity of emergency medical suppliers based on flexible contracts. This approach offers a scientific foundation for the government to effectively address the challenge of supplier quantity selection when faced with risks related to shortages, expiration, and the combination of both.

## 1 Introduction

The proper selection of emergency medical suppliers plays a crucial role in ensuring effective government-enterprise joint reserve efforts. A reliable supplier network significantly reduces the risk of supply disruptions. To mitigate the risks associated with shortages or medical supply expiration (supply risk), the government typically adopts a strategy of multiple joint purchases and reserves for various emergency medical supplies (1, 2). Additionally, the government takes into account various factors such as supplier capacity (e.g., supply quantity, stability of continuous supply, and responsiveness), reputation, and the scale of disasters (3). In order to address the uncertainties of emergency medical supplies demand, the government proactively establishes flexible contracts with multiple suppliers (4). These contracts feature adjustable parameters such as purchase quantity, price, incentive levels, and constraint conditions. This approach not only helps prevent the risks of medical shortages and shelf-life issues, but also reduces the cost of government procurement and storage management, while compensating suppliers for potential losses associated with flexible inventory. Medical responsiveness depends on two key aspects: first, the urgency of medical needs (5). The higher the urgency of victim needs, the greater the priority for medical response. Second, the response characteristics of government-enterprise reserves (6). As emergency rescue is a critical governmental function, it possesses real-time monitoring, response, and disposal systems for medical scheduling, resulting in relatively high reserve responsiveness.

Based on the aforementioned analysis, this paper will consider the selection of the number of suppliers from three dimensions: the supply risk of emergency medical supplies (with a particular focus on stock shortage and shelf-life risks), the reserve cost of government procurement, and the responsiveness of emergency medical supplies. In other words, by emphasizing supply risk and taking into account the cost and reserve considerations of government procurement, as well as the responsiveness of emergency medical supplies (including the response level of emergency medical supplies and the characteristics of government and enterprise reserves), this study aims to determine the appropriate number of suppliers that can effectively address potential disruptions in medical supply. This paper introduces the concepts of call options, put options, and two-way options from supply chain contract theory into government-enterprise joint reserve agreements. By coordinating different flexible contracts under various scenarios, the study aims to scientifically adjust contract parameters, including procurement quantity, medical price, incentive level, and constraint conditions, between the government and enterprise. The focus is on investigating the rational selection of the number of government emergency medical suppliers, addressing the following three specific sub-problems:

1. Quantity selection of call option contract suppliers considering stock shortage risk: in cases where emergency medical supplies are susceptible to stock shortages, the government enters into call option contracts with suppliers. This approach aims to minimize government costs during the reserve period while maximizing supplier benefits and reducing the risk of stock shortages.
2. Number selection of put option contract suppliers considering shelf-life risk: emergency medical supplies with limited shelf-life may face risks related to expiration. To avoid medical waste and address the issue of suppliers assuming excessive risks, the government can establish put option contracts with suppliers.
3. Two-way option contract supplier quantity selection problem, considering the coexistence of stock shortage risk and shelf-life risk: emergency medical supplies face the dual risks of stock shortages and shelf-life limitations. Demand for these supplies may be either zero or significantly exceed the suppliers’ capacity to meet it. Introducing two-way option contracts enables the government to effectively manage demand fluctuations of emergency medical supplies. This approach facilitates short-term, efficient, and sufficient supply of emergency medical supplies, improves the buyback guarantee for emergency medical supplies by suppliers, and compensates suppliers for the additional risks they assume.

## 2 Literature review

Emergency medical supplies are essential resources needed throughout the entire emergency management process in response to major natural disasters, accidents, public health crises, or social security emergencies (7). These emergency medical supplies possess characteristics of uncertainty, irreplaceability, timeliness, and hysteresis. The government’s reserve capacity is limited. During large-scale natural disasters, government medical reserves may fall short in meeting the demand, thereby facing the risk of shortages (8). On the other hand, if disasters do not occur within the reserve cycle, there is a risk of medical waste due to expiration. Agreed-upon enterprise reserves possess scale advantages, ensuring the effective circulation and dynamic replenishment of emergency medical supplies. However, the responsiveness and reliability of enterprise inventories are relatively weak. Therefore, scholars have proposed the concept of government-enterprise joint reserves, known as “joint reserves of government and enterprise” (9). The joint reserve of government and enterprise effectively compensates for their respective weaknesses while leveraging their individual strengths to establish a reliable and efficient emergency medical supplies reserve. From a market mechanism perspective, government-enterprise joint reserves can achieve win-win outcomes and foster sustainable and stable development for both the government and suppliers (10). Compared to separate government reserves, the government-enterprise joint reserve mode reduces government storage costs and alleviates the pressure on the emergency medical supplies supply chain. Simultaneously, enterprises benefit from reserve income and government subsidies. In the event of emergencies, enterprises can also increase their medical sales volume and improve overall sales performance (11, 12).

### 2.1 The coordination of supply chain contracts in government-enterprise joint reserve

Supply chain contract coordination theory has been widely applied to address the challenges of government-enterprise joint emergency medical supplies reserve and supply, resulting in significant research findings. To mitigate the shortage of emergency medical supplies under uncertain circumstances, option contracts can be utilized. Option contracts allow buyers to determine the purchase quantity based on their specific needs by paying a certain option cost, thereby hedging the risks associated with demand fluctuations. Scholars have explored various flexible contract mechanisms, such as quantity flexible contracts, which enable deviations from pre-ordered quantities within a certain range, thereby improving the quantity of emergency medical supplies while considering cost control and supplier income realization (11, 12). Call option contracts have also been proposed, where the buyer and supplier agree in advance that in the event of medical shortages, the buyer can purchase emergency medical supplies at a predetermined price by paying an option cost to the supplier. This contract effectively alleviates stock shortage risks during emergency medical supplies procurement (13). While both quantitative flexible contracts and call option contracts address the uncertainty of emergency medical supplies demand to some extent, call option contracts are more targeted in mitigating medical shortage risks and motivate enterprises through payment rights. Therefore, this paper focuses on the use of call option contracts to analyze and study the selection of suppliers under shortage risks.

Regarding the shelf-life risk of emergency medical supplies, repurchase contracts have been introduced. These contracts allow enterprises to repurchase unused emergency medical supplies from the government at an agreed price. Repurchase contracts help prevent waste of emergency medical supplies held in government reserves when disasters do not occur (14). By signing repurchase contracts, the government can reduce its losses, while enterprises can recover emergency medical supplies nearing expiration at a discounted rate and reintroduce them into the market based on the remaining shelf-life’s discount rate. This arrangement benefits both the enterprise and compensates for the risks it assumes (15). Some scholars have also introduced put option contracts, which possess the characteristics of options, into emergency medical supplies procurement and reserve models. Put option contracts help avoid losses caused by shelf-life risks, analyze the coordination mechanism between government and enterprise supply chains, and address the issue of suppliers bearing redundant risks in traditional supply chains (16). In summary, repurchase contracts and put option contracts alleviate the uncertainty of emergency medical supplies demand timing and effectively handle shelf-life risks. Put option contracts, in particular, compensate suppliers for the risks they bear, leading to a win-win situation between the government and suppliers.

In the case of uncertain natural disasters, emergency medical supply availability often faces both shortage risks and shelf-life risks. Some scholars propose the use of two-way option contracts for coordination which considers the characteristics of both bullish and bearish options. Studies have shown that these contracts effectively reduce government stock shortage risks and shelf-life risks when considering the probability of emergency events (17). Additionally, the combination of two-way option contracts and B2B electronic markets can effectively handle demand fluctuations caused by emergencies (18). It is evident that in emergency fields, supply chain coordination using two-way option contracts based on demand uncertainty offers greater flexibility and can better address shortage risks and shelf-life risks. However, most studies on supplier selection under two-way option contracts do not consider the number of options.

### 2.2 The selection of the quantity of emergency medical suppliers

Supplier quantity selection is closely linked to supply chain disruption (19). Several studies have addressed this issue by developing optimization models and decision-making approaches. Decision tree model that considers utility value and probability as variables to optimize the supplier quantity under risks and uncertainties. They discussed the conditions and parameters where a multi-supplier strategy outperforms a single-supplier strategy, and explored the optimal supplier quantity based on specific numerical calculations (20). Darvazeh designed an integer programming model to determine the optimal number of suppliers by considering the quantity elastic contract, supply risk randomness, and market demand uncertainty, with the goal of maximizing the benefits of supply chain enterprises (21). The supply risk of suppliers and the demand risk of the market to design an optimal quantity decision model for suppliers, aiming to minimize the total procurement cost while considering the risk of supply chain disruption (22). Optimized the quantity selection of suppliers using economic batch methods or mathematical programming methods. They established a quantity optimization model within the purchaser’s acceptable range, demonstrating that the optimal supplier quantity can be obtained by combining supply risk, procurement cost factors, and random market conditions (23). Zhang Zhixiang compared the advantages and disadvantages of single-source supply and multi-source supply, high-lighting that the risk associated with multi-source supply is significantly lower. They proposed a model based on the principle of the lowest total procurement cost, where the purchaser must balance cost reduction and supply risk while including the loss caused by supply risk in the total procurement cost (24). The adequate supply of emergency medical supplies is vital for coping with the risk of supply chain disruption and ensuring the lives, health, and safety of victims. Supplier quantity selection involves a trade-off between minimizing expected disruption losses and maximizing supplier utility based on criteria such as cost and flexibility (25).

Although research on the quantity selection of emergency medical suppliers is available, few studies consider the influence of multiple scenario factors on the decision-making process, and mathematical planning methods may have limitations in reflecting quantity flexibility. In emergency situations, the demand for emergency medical supplies is difficult to determine, and the interaction of multiple scenario factors plays a critical role in supplier quantity selection. Therefore, it is necessary to further consider the influence of multiple scenario factors when optimizing the quantity decision in flexible contract coordination. The study will consider multiple scenario factors, including the supply risk of emergency medical supplies, the cost of government procurement reserve, and the responsiveness of emergency medical supplies.

## 3 Medicals and methods

### 3.1 Emergency medical suppliers selection

When evaluating and selecting emergency medical suppliers, it is important to define the characteristics of emergency medical supplies in order to make informed judgments. The nature of emergency medical supplies, shaped by the uncertainty of sudden disasters, encompasses the following key aspects: (1) Uncertainty: the occurrence of natural disasters and emergencies brings about uncertainty regarding the timing, location, intensity, and impact scope. (2) Irreplaceability: emergency medical supplies differ from general supplies as they are specially designed for use in specific environments (26). (3) Timeliness: timeliness stands out as a critical feature of emergency medical supplies. These supplies must be swiftly transported to disaster sites within a designated timeframe. (4) Time lag: the inherent nature of emergencies, characterized by their unexpected occurrence, makes it impossible to anticipate the precise timing and location of disasters in advance. In summary, the evaluation index system of supplier selection as Table 1.

**Table 1:** Evaluation index of supplier selection.

First grade index	Second grade index	Type of index	Index definition
Medical evaluation	Percent of pass	Quantitative	Whether the medical quality meets the legal requirements
	Refund rate	Quantitative	Medicals returned due to quality problems
	Price rigidity	Quantitative	The amount of fluctuation over a period of time
Service quality evaluation	Punctuality	Quantitative	Suppliers deliver on time according to the stipulated time
	Breakage rate	Quantitative	Whether the medical be in good condition after transportation
	Error rate	Quantitative	Whether the medical meets the standard
	After-sales service	Qualitative	Whether solve the damage quickly and effectively
Emergency capacity evaluation	Medicalion capacity	Quantitative	Equipment utilization rate, daily medicalivity
	Transfer capability of medicalion line	Qualitative	The conversion capacity of the medicalion line to produce different medicals
	Emergency order capacity	Qualitative	Ability to contact medical supplier for urgent order at short notice
	Emergency distribution capacity	Qualitative	The ability to expedite the delivery of emergency medical supplies
Synthetically corporate evaluation	Corporate image	Qualitative	Public reputation
	Business prospects	Qualitative	Enterprise development opportunities and market prospects
	Geographic position	Qualitative	The transportation development degree of the enterprise location
	Information level	Qualitative	The degree of enterprise informatization

These indexes should be closely linked to emergency operations and emphasize the suppliers’ ability to effectively respond to emergencies. The aim is to ensure that selected suppliers can produce high-quality emergency medical supplies within the shortest possible time, meeting the necessary specifications, and facilitating the smooth progress of emergency rescue efforts.

Selection of emergency medical suppliers which involves determining an appropriate number of suppliers that can prevent disruptions in the provision of emergency medical supplies and ensure their reliability. Having too many suppliers can reduce supply risks but increases the cost associated with selecting and managing government suppliers. Conversely, having too few suppliers reduces option and management costs but compromises the reliability of emergency medical supplies (27). Therefore, finding a balance between reducing supply risks and controlling reserve costs is crucial, and careful attention should be given to the quantity selection of emergency medical suppliers. Currently, there are two main management approaches for selecting the number of suppliers: the single supplier strategy and the multi-supplier strategy. Studies on optimizing supplier quantity selection primarily focus on utility value and risk balance, employing decision tree models, integer programming, economic batch methods, and other techniques. Given the numerous factors influencing the government’s selection of suppliers, the analysis of different types of emergency medical supplies should be conducted on a case-by-case basis.

In this study, the government’s approach begins by establishing an evaluation index system for supplier selection, which enables the identification of available suppliers based on emergency medical suppliers selection principles. While addressing the uncertainty of medical demand, the research aims to consider scenario factors such as the supply risk of emergency medical supplies and the responsiveness of emergency medical supplies. The goal is to minimize the reserve costs associated with government procurement.

### 3.2 Contract coordination theory of supply chain

#### 3.2.1 Coordination of call option contracts

**Figure 1:**
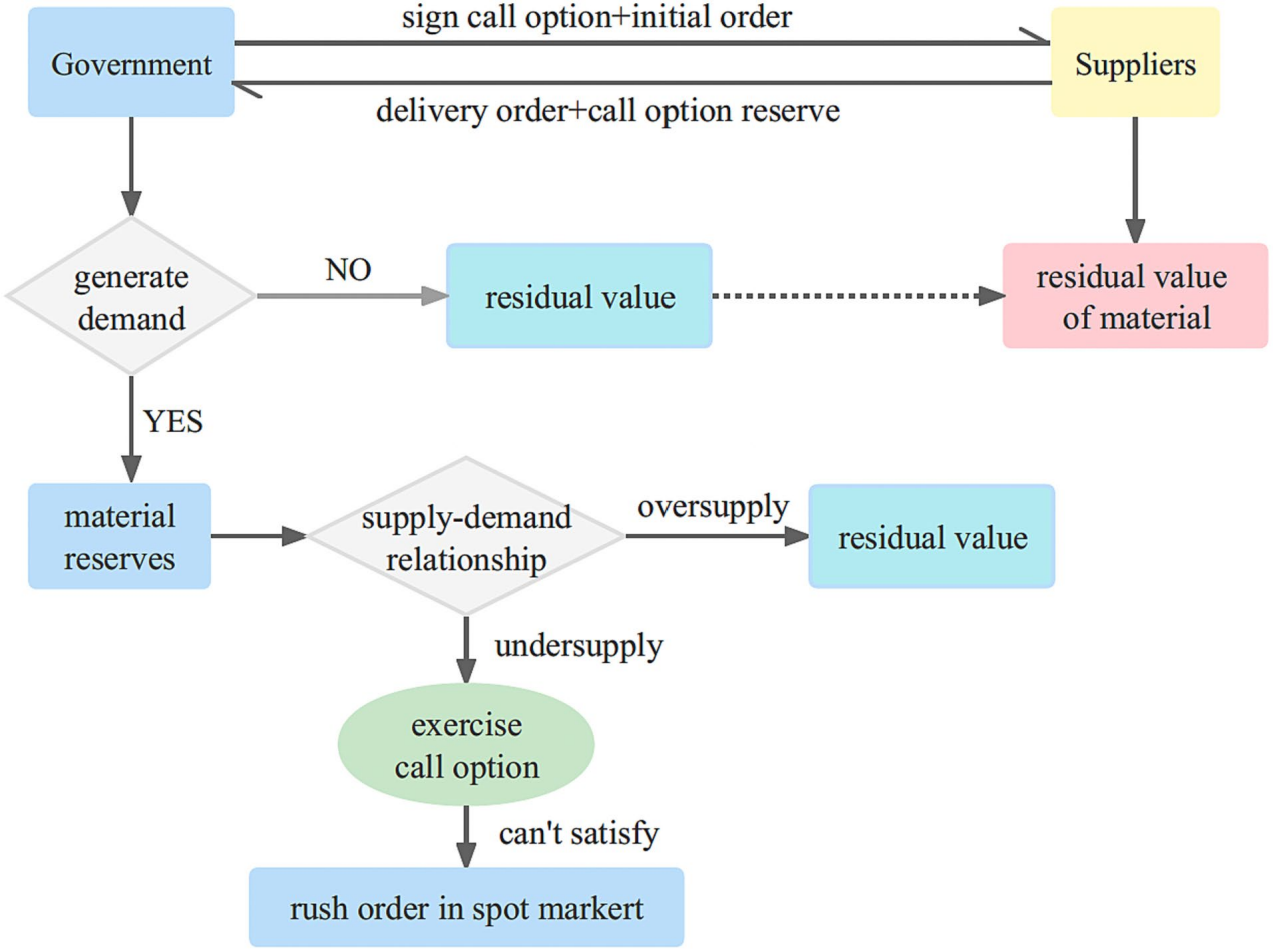
Call option contract model of government-enterprise cooperation.

During the reserve period, the government prioritizes the use of its own stockpile of emergency medical supplies. However, in the event that the government’s inventory is insufficient to meet the demand during an emergency, the call option can be exercised. This allows the government to procure additional physical emergency medical supplies from the supplier, up to the predetermined quantity, at an agreed-upon price. As part of the contract, the government pays an option fee to the supplier when the option is invoked. The utilization of call option contracts alleviates the pressure on the government’s inventory, as it allows them to supplement their reserves by procuring emergency medical supplies from external suppliers. This helps reduce inventory costs and mitigates the risk of stock shortages. The implementation of a single government-single enterprise single cycle emergency medical supplies contract model as Figure 1.

1. The government initiates the procurement process by purchasing a specific unit of physical emergency medical supplies from the supplier, which is then stored in the government repository. Simultaneously, a call option contract is entered into with the supplier. This contract stipulates that the government has the option to purchase an additional unit of option emergency medical supplies from the supplier at a predetermined price, which is typically lower than the prevailing market price. As part of the agreement, the government pays an option cost to the supplier.
2. During the reserve period, if the government’s physical inventory falls short of meeting the demand for emergency medical supplies, it can exercise the call option and purchase additional emergency medical supplies from the supplier, up to the agreed quantity, at the predetermined call option price. However, if the demand still exceeds the available inventory, the government can resort to emergency purchases from the spot market at market prices.
3. At the conclusion of the reserve period, if there is no occurrence of an emergency or if the government has surplus inventory, it takes responsibility for managing the excess emergency medical supplies and receives the corresponding salvage value income. Conversely, the supplier receives the salvage value associated with the agreed-upon purchase quantity.

This emergency medical supplies procurement and reserve mode alleviates the pressure on the government’s emergency medical supplies reserve, reducing the associated costs. Moreover, from the perspective of suppliers which increases their overall profitability.

#### 3.2.2 Coordination of put option contract

Unlike the call option contract, where the government has the right to purchase a specified quantity of emergency medical supplies from the supplier at an agreed price, the put option contract allows the government to require the supplier to repurchase any remaining emergency medical supplies, up to the agreed amount, at the predetermined price once the contract period concludes. This arrangement serves multiple purposes: it enables the government to minimize its costs, avoids the waste of expired emergency medical supplies, and compensates the supplier for the risks they undertake by providing additional royalties. As a result, a mutually beneficial outcome is achieved for both the government and the enterprise.

**Figure 2:**
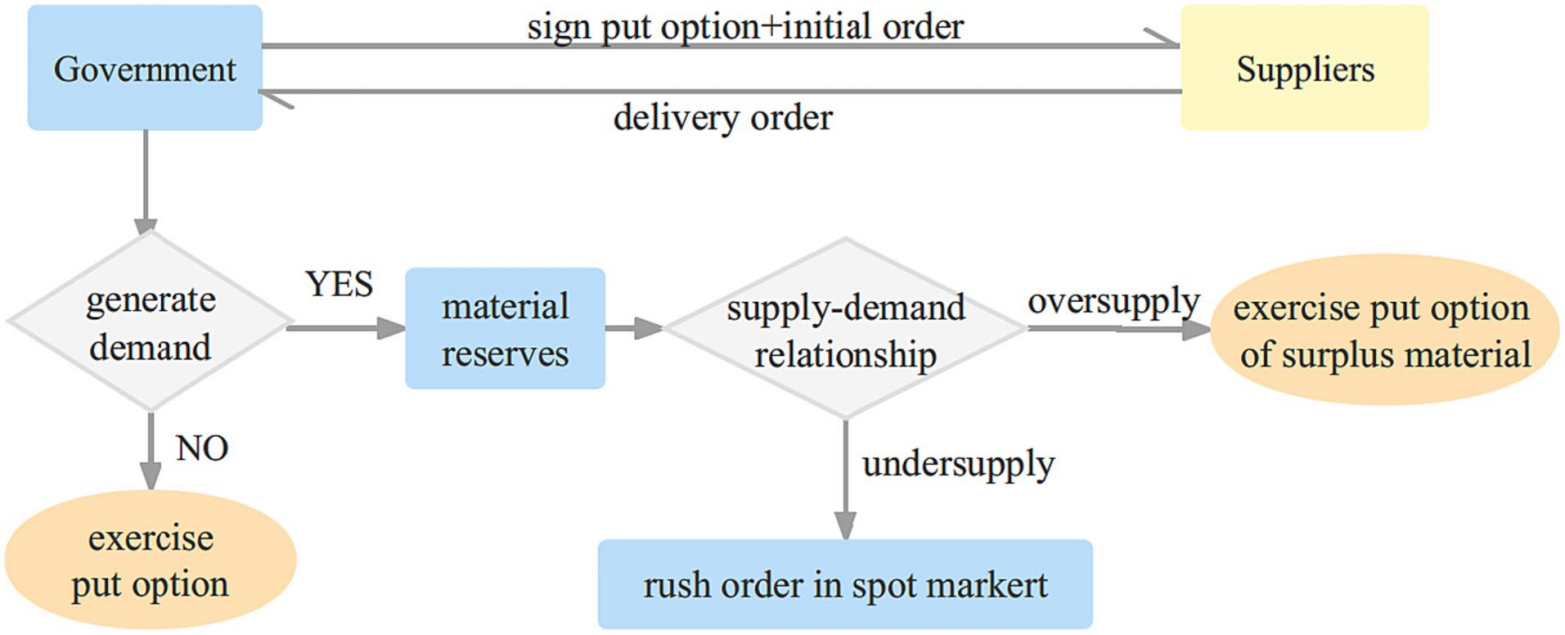
Put option contract model of government-enterprise cooperation.

Given that emergencies often have a low probability of occurrence and certain emergency medical supplies possess a short shelf-life, the government can opt to enter into put option contracts with enterprises. By doing so, the government mitigates the risk of waste from expired emergency medical supplies in the future while sharing the risk burden with the enterprises. This arrangement enables a collaborative risk management approach and alleviates the risk pressure on the government. The put option contract model for single government-single enterprise single cycle emergency medical supplies is illustrated in Figure 2.

1. The government procures a specific quantity of physical emergency medical supplies from the supplier to be stored in the government repository. Additionally, a put option contract is established between the government and the supplier. This contract allows the government to require the supplier to repurchase any surplus emergency medical supplies at a predetermined price, while compensating the supplier with an option cost.
2. Throughout the reserve period, if a disaster occurs and the government’s physical inventory falls short, the government has the option to make emergency purchases from the spot market at prevailing market prices.
3. At the conclusion of the reserve period, in cases where no emergency arises or there is surplus inventory remaining, the government can exercise the option to sell the excess emergency medical supplies back to the supplier at the predetermined put option strike price. The supplier will then handle the emergency medical supplies and benefit from the residual income.

The adoption of a put option contract as the basis for emergency medical supplies procurement and reserve which effectively addresses the risk of medical wastage due to expiration, reduces government costs, and provides appropriate compensation to suppliers for assuming additional risks. This approach ensures the protection of supplier interests and fosters a mutually beneficial relationship between the government and the supplier.

#### 3.2.3 Coordination of two-way option contracts

**Figure 3:**
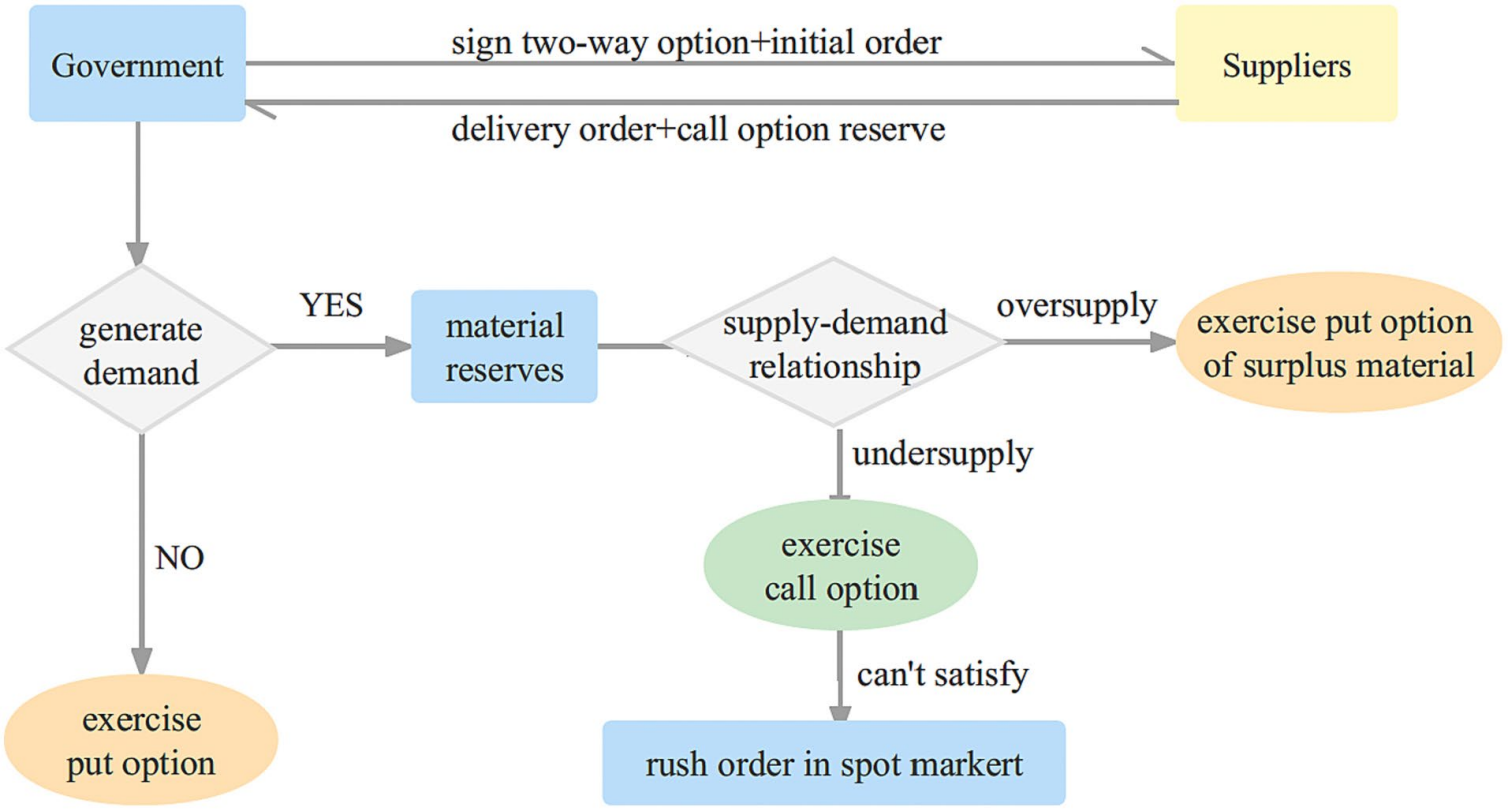
Two-way option contract model of government-enterprise cooperation.

Two-way options are related to medical shelf-life and stock shortages, has received limited research attention. Integrating two-way options can effectively mitigate these dual risks and ensure the smooth execution of emergency rescue operations. Figure 3 illustrates the joint government-supplier reserve mode under the two-way option framework.

1. Reserve options: to effectively address emergencies, the government procures a specific quantity of emergency medical supplies from suppliers, which are then stored in the emergency reserve pool. Simultaneously, both parties enter into a two-way option agreement, outlining the purchase of a certain number of real call options and put options by the government from the supplier throughout the contractual period.
2. During the reserve period, priority is given to utilizing the government’s physical reserves, followed by the use of options. In situations where the government’s emergency reserves prove insufficient, the call option can be exercised to procure physical emergency medical supplies from the supplier, up to the agreed-upon quantity. If the demand still cannot be fully met, the government may need to resort to spot purchases, and selectively exercise call options to mitigate the risk of medical shortages.
3. In the end of the reserve period, any surplus emergency medical supplies held in the government’s physical reserves can be repurchased by the supplier at the predetermined put option strike price. The supplier can then handle the surplus emergency medical supplies and realize the corresponding benefits.

The adoption of a two-way option contract not only mitigates the risk of medical supply expiration for the government but also effectively addresses the uncertain demand for emergency medical supplies and manages the risk of medical shortages. In summary, the coordination of contract theory can be applied in various scenarios, as Table 2.

**Table 2:** Option contract coordination of supply chain.

Contract nature	Usage scenario
Call option contract	At risk of out-stock, government reserves are in short supply
Put option contracts	At risk of pass expiration date, the government reserve oversupply
Two-way option contract	Both two types of risk

### 3.3 The supplier selection model

In the selection of emergency medical suppliers, there are several costs involved:

1. Option transaction costs. The government incurs direct transaction costs while establishing a joint reserve relationship with suppliers. As the number of suppliers decreases and the government becomes more dependent on them, there is a risk of opportunistic behavior. Suppliers may take advantage of the government’s dependence and trust to seek improper benefits, resulting in opportunistic costs for the government.
2. Loss from shortage. Shortage risk refers to delays in delivery time, quantity shortages, and quality defects caused by suppliers. To mitigate supply chain shortage risk, call option contracts can be utilized. If there is a shortage of emergency medical supplies in the government’s emergency stockpile, the government can exercise the call option to purchase emergency medical supplies from the supplier. If the reserves are insufficient to meet the demand, the government procures emergency medical supplies from the spot market at market purchase prices. At the end of the reserve period, if there is surplus medical in the government’s reserve, the government can dispose of it and obtain corresponding income. If the government chooses not to exercise the call option, the supplier can obtain the salvage value of the medical.
3. Emergency procurement cost. This cost refers to the amount paid by the government to purchase additional emergency medical supplies from suppliers during emergencies. The procurement cost can vary depending on three situations: when the sum of government reserve and government-enterprise option reserve is still insufficient to meet the emergency demand, when the emergency demand exceeds the sum of reserves, and when the emergency demand is lower than the government reserve.
4. Income from residual value of shelf-life. Shelf-life risk relates to the surplus of emergency medical supplies in the government warehouse due to the absence or small scale of disasters. The government may generate income by selling the surplus emergency medical supplies or utilizing them in other ways.

#### 3.3.1 Theoretical framework: supplier selection for call option contracts

**Figure 4:**
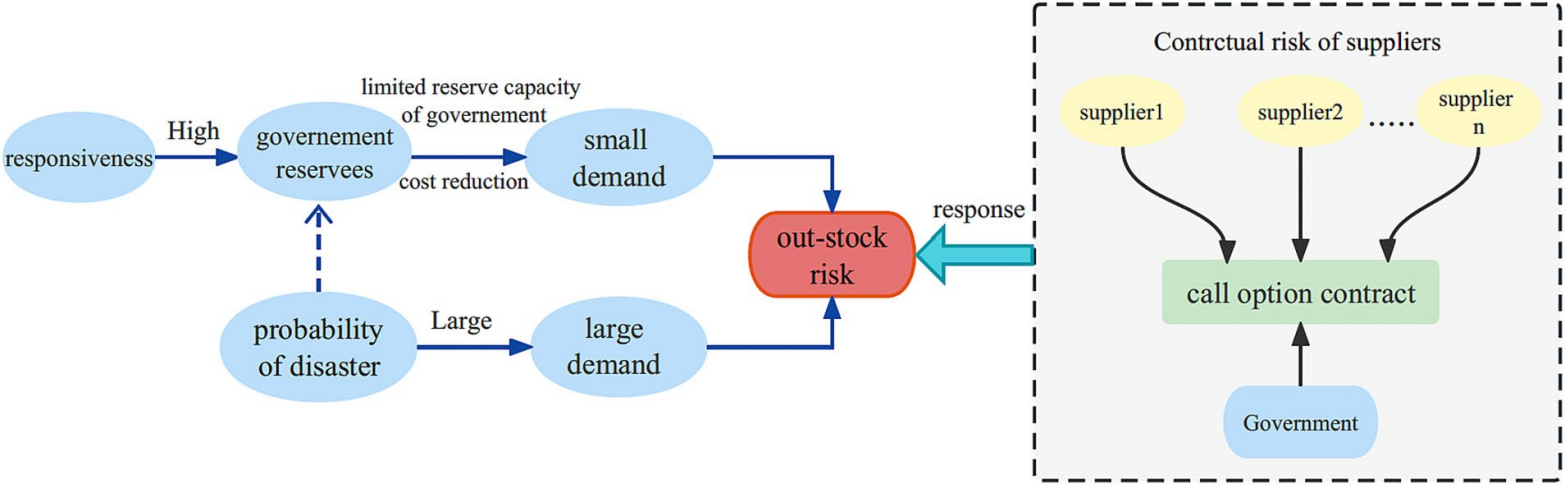
Theoretical framework of supplier selection for call option contracts.

Emergency medical suppliers operating in the market face inherent performance risks, such as supplier default, or disruptions in supply caused by unforeseen emergencies. To ensure a consistent supply of emergency provisions, it becomes imperative to engage multiple emergency medical suppliers. By determining the optimal number of emergency medical suppliers, not only can the reliable provision of emergency medical supplies be ensured, but also government procurement costs can be minimized, thereby alleviating the strain on government reserves. The theoretical framework depicting the process of supplier selection for call option contracts is illustrated in Figure 4.

In scenarios where emergency medical supplies are in high demand, there is a potential risk of shortages. The call contract stipulates a predetermined unit option fee and associated option costs, which are paid by the government. Simultaneously, the supplier establishes a reserve of call options specifically for the government’s utilization whenever necessary. Throughout the contract duration, if an unforeseen disaster occurs or there is a surge in demand, the government has priority access to the reserved emergency medical supplies before resorting to the use of options. In the event that the government’s emergency reserve falls short, it has the authority to exercise the call option and procure physical emergency medical supplies from the supplier, as long as the quantity remains within the agreed limit. Conversely, if there is no demand or an excess of emergency medical supplies in the government’s stockpile, the surplus is appropriately managed to retrieve salvage value. Which alleviates the burden of maintaining excessive reserves for the government while minimizing associated costs. And it ensures a more reliable supply of emergency medical supplies, thereby enhancing the government’s ability to respond effectively to crises (Table 3).

1. The probability that i suppliers will supply medicals normally, where(0 ≤ *i* ≤ *n*), is $C_{n}^{i}p^{n-i}{\left(1-p\right)}^{i}$.
2. The probability that the government’s reserve will be insufficient to meet the emergency demand is $C_{n}^{i}p^{n-i}{\left(1-p\right)}^{i}.p\left(Q_{u}+q_{u}i\leq \xi \leq Q_{u}+q_{u}n\right)$.
3. When the joint reserve of the government and the enterprises under the contract fails to meet the emergency demand, the government must purchase goods at market prices, and the expected loss due to out-of-stock for the government is $C_{n}^{i}p^{n-i}{\left(1-p\right)}^{i}\overset{U}{\underset{Q_{u}+q_{u}i}{\int }}r\left[\xi -\left(Q_{u}+q_{u}i\right)\right]f\left(\xi \right)d_{\xi }$.
4. When the government’s initial reserves meet the emergency demand, the procurement cost is zero.
5. $Q_{u}+q_{u}$i<ξ, when the sum of the government’s initial reserve and the government-enterprise option reserve is still less than the emergency demand.
6. $Q_{u}+q_{u}$i$\geq \xi >Q_{u}$, when the emergency demand exceeds the sum of the government’s initial reserve and the government-enterprise option reserve.
  The call option model is formulated as follows: Equations 1.1–1.8.

(1.1) $$TC\left(n\right)\;=\;PQ_{u}+Uu\;+\;Vu\;+\;Wu$$

(1.2) $$\text{Uu}=a+{\text{bn}}^{\text{θ}}+\text{c}/\text{n}+\text{o}q_{u}n$$

(1.3) $$V_{u}=\overset{n}{\underset{i=0}{\sum }}C_{n}^{i}p^{n-i}{\left(1-p\right)}^{i}\overset{U}{\underset{Q_{u}+q_{u}i}{\int }}r\left[\xi -\left(Q_{u}+q_{u}i\right)\right]f\left(\xi \right)d_{\xi }$$

(1.4) $$\overset{n}{\underset{i=0}{\sum }}C_{n}^{i}p^{n-i}{\left(1-p\right)}^{i}\overset{U}{\underset{Q_{u}+q_{u}i}{\int }}\omega_{u}q_{u}\mathrm{if}\left(\xi \right)d_{\xi }$$

(1.5) $$\overset{n}{\underset{i=0}{\sum }}C_{n}^{i}p^{n-i}{\left(1-p\right)}^{i}\overset{Q_{u}+q_{u}i}{\underset{Q_{u}}{\int }}\omega_{u}\left(\xi -Q_{u}\right)f\left(\xi \right)d_{\xi }$$

(1.6) $$\begin{array}{l} W_{u}=\overset{n}{\underset{i=0}{\sum }}C_{n}^{i}p^{n-i}{\left(1-p\right)}^{i}\\ \left\{\overset{U}{\underset{Q_{u}+q_{u}i}{\int }}\omega_{u}q_{u}\mathrm{if}\left(\xi \right)d_{\xi }+\overset{Q_{u}+q_{u}i}{\underset{Q_{u}}{\int }}\omega_{u}\left(\xi -Q_{u}\right)f\left(\xi \right)d_{\xi }\right\} \end{array}$$

(1.7) $$\begin{array}{c} TC\left(n\right)=PQ_{u}+U_{u}+V_{u}+W_{u}=PQ_{u}+a+bn^{\theta }\\ +c/n+oq_{u}n+\overset{n}{\underset{i=0}{\sum }}C_{n}^{i}p^{n-i}{\left(1-p\right)}^{i}\\ \left\{\begin{array}{l} \overset{U}{\underset{Q_{u}+q_{u}i}{\int }}r\left[\xi -\left(Q_{u}+q_{u}i\right)\right]f\left(\xi \right)d_{\xi }+\overset{U}{\underset{Q_{u}+q_{u}i}{\int }}\omega_{u}q_{u}if\left(\xi \right)d_{\xi }\\ +\overset{Q_{u}+q_{u}i}{\underset{Q_{u}}{\int }}\omega_{u}\left(\xi -Q_{u}\right)f\left(\xi \right)d_{\xi } \end{array}\right\} \end{array}$$

(1.8) $$s.t.=\{\begin{array}{c} n\geq 1\\ n\in Z \end{array}$$

**Table 3:** Symbols.

Variable	Definition	Variable	Definition
*n*	The number of emergency medical suppliers	$\omega_{u}$	The exercise price of call option contract
*TC(n)*	Total cost	$\omega_{d}$	The exercise price of put option contract
*P*	Initial unit price of emergency medical supplies purchased by the government	$Q_{u}$	Medical stocking quantity of call option contract
*p*	Supplier default risk probability	$Q_{d}$	Medical stocking quantity of put option contract
*o*	Unit cost on call, put and two-way options	*Q*	Medical stocking quantity of two-way option contract
*v*	Unit loss value of emergency medical supplies	$q_{u}$	Purchase quantity for single supplier of call option contract
*r*	nit purchase price of expected market	$q_{d}$	Purchase quantity for single supplier of put option contract
ξ	Random demand for emergency medical supplies, *F(*ξ*)* is *probability distribution function*, f*(*ξ*) is probability density function*, mean is μ, variance is $\;\sigma^{2}$, Gaussian distribution$\xi \sim \left(\mu \text{,}\sigma^{2}\right)$, maximum is $U(U\leq Q_{u}$*+*$q_{u}n$)	$V_{d}$	Shelf-life residual value
*Uu*	Transaction cost of call option contract	*Wu*	Emergency procurement cost
$U_{d}$	Transaction cost of put option contract	*a*	Fixed transaction costs, constant
$U_{n}$	Transaction cost of two-way option contract	*b*	Increase the direct transaction cost of a supplier, constant
*Vu*	Shortage costs	*θ*	Cooperation degree, constant

*P*$Q_{u}$ is the initial government reserve procurement cost. The opportunistic cost for the government is *c*/*n*, which decreases as the number of suppliers increases. The government pays an option fee *o*$q_{u}$ to acquire $q_{u}$ call option medicals from a supplier.

#### 3.3.2 Theoretical framework for supplier selection of put option contracts

**Figure 5:**
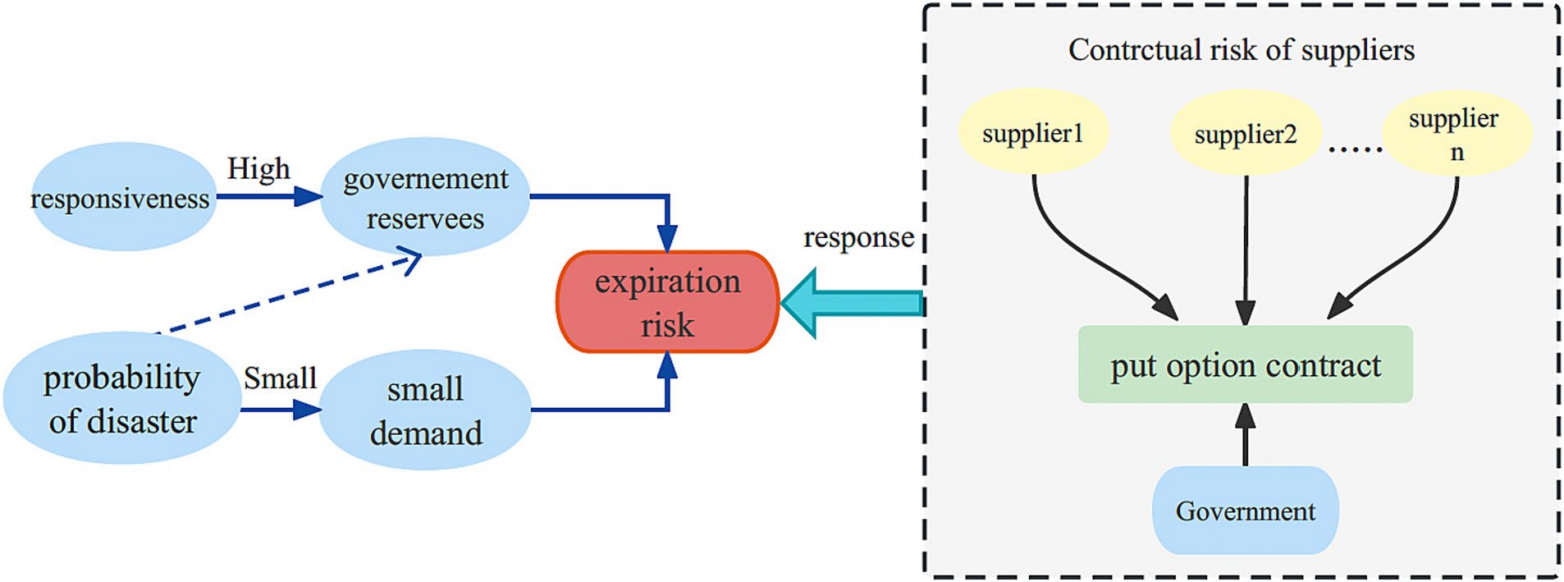
Theoretical framework of supplier selection for put option contracts.

Emergency medical supplies reserved by the government often have high responsiveness and shelf-life requirements, making them susceptible to the risk of expiration, leading to resource wastage. It can easily expire due to long-term storage, resulting in both medical waste and increased financial burden for the government. To mitigate this risk, the government can enter into put option contracts with suppliers, effectively addressing the shelf-life risk associated with emergency medical supplies. Additionally, given the performance risk of emergency medical suppliers in the market, a systematic selection process for the number of emergency medical suppliers is crucial to avoid resource waste and reduce government costs. This approach promotes a mutually beneficial outcome for both the government and the suppliers, fostering a win-win situation. The theoretical framework for the supplier selection of put option contracts is illustrated in Figure 5.

Procurement cost of the government's option reserve is *P*$Q_{d}$.

1. In scenarios where there are i suppliers involved in normal buyback, the probability that the government’s reserves exceed the emergency demand is given by $C_{n}^{i}p^{n-i}{\left(1-p\right)}^{i}.p\left(0\leq \xi \leq Q_{d}\right)$. When surplus medicals are still present in the government’s reserve pool under the put option contract, the government can independently handle the emergency medicals and obtain corresponding surplus value income. During this time, the unit loss incurred by the government is v, while the unit surplus value obtained from the medicals is $\left(P-v\right)$.
2. When the demand for emergency medicals is not lower than the government’s initial reserve, the residual value of shelf-life is zero.
3. When the demand for emergency medicals is less than the difference between the government’s initial reserve and the government-enterprise option reserve, $0\leq \xi <Q_{d}-q_{d}i$.
4. When the demand for emergency medicals is not less than the difference between the initial reserve and the government-enterprise option reserve, but smaller than the initial government reserve, $Q_{d}-q_{d}i\leq \xi <Q_{d}$
  The put option model is formulated as follows: Equations 2.1–2.7.

(2.1) $$TC\left(n\right)=PQ_{d}+U_{d}-V_{d}$$

(2.2) $$Ud=a+bn^{\theta }+c/n+oqdn$$

(2.3) $$\overset{n}{\underset{i=0}{\sum }}C_{n}^{i}p^{n-i}{\left(1-p\right)}^{i}\overset{Q_{d}-q_{d}i}{\underset{0}{\int }}\left[\left(Q_{d}-q_{d}i-\xi \right)\left(P-v\right)+\omega_{d}q_{d}i\right]f\left(\xi \right)d_{\xi }$$

(2.4) $$\overset{n}{\underset{i=0}{\sum }}C_{n}^{i}p^{n-i}{\left(1-p\right)}^{i}\overset{Q_{d}}{\underset{Q_{d}-q_{d}i}{\int }}\left(Q_{d}-\xi \right)\omega_{d}f\left(\xi \right)d_{\xi }$$

(2.5) $$\begin{array}{c} V_{d}=\overset{n}{\underset{i=0}{\sum }}C_{n}^{i}p^{n-i}{\left(1-p\right)}^{i}\\ \left\{\begin{array}{c} \overset{Q_{d}-q_{d}i}{\underset{0}{\int }}\left[\left(Q_{d}-q_{d}i-\xi \right)\left(P-v\right)+\omega_{d}q_{d}i\right]f\left(\xi \right)d_{\xi }\\ +\overset{Q_{d}}{\underset{Q_{d}-q_{d}i}{\int }}\left(Q_{d}-\xi \right)\omega_{d}f\left(\xi \right)d_{\xi } \end{array}\right\} \end{array}$$

(2.6) $$\begin{array}{l} TC\left(n\right)=PQ_{d}+U_{d}-V_{d}=PQ_{d}+a+bn^{\theta }+\frac{c}{n}+oq_{d}n\\ -\overset{n}{\underset{i=0}{\sum }}C_{n}^{i}{\begin{array}{c} p^{n-i}\\ \left(1-p\right) \end{array}}^{i}\left\{\begin{array}{c} \overset{Q_{d}-q_{d}i}{\underset{0}{\int }}\left[\left(Q_{d}-q_{d}i-\xi \right)\left(P-v\right)+\omega_{d}q_{d}i\right]f\left(\xi \right)d_{\xi }\\ +\overset{Q_{d}}{\underset{Q_{d}-q_{d}i}{\int }}\left(Q_{d}-\xi \right)\omega_{d}f\left(\xi \right)d_{\xi } \end{array}\right\} \end{array}$$

(2.7) $$s.t.=\{\begin{array}{c} n\geq 1\\ n\in Z \end{array}$$

If there is no demand for emergency medical supplies due to sudden disasters or if the demand is low and there are remaining emergency medical supplies in the government’s emergency reserve pool, the government can request the supplier to repurchase the surplus emergency medical supplies, up to the agreed quantity, at the strike price stipulated in the put option contract. This way, the government not only reduces its own costs and prevents the wastage of expired emergency medical supplies but also compensates the risks borne by the enterprises, achieving a mutually beneficial outcome for both parties.

#### 3.3.3 Theoretical framework for supplier selection of two-way option contracts

**Figure 6:**
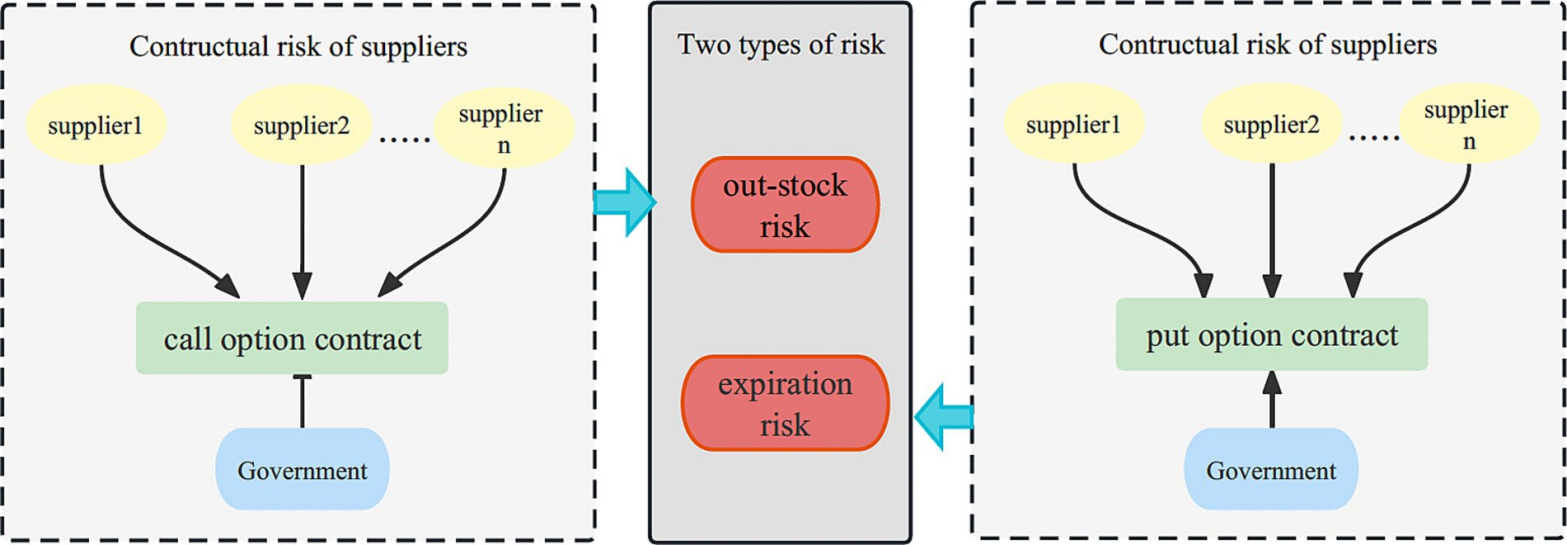
Theoretical framework of supplier selection for two-way option contracts.

In situations where emergency medical supplies face both the risk of shortages and the risk of expiration, it is necessary for the government to enter into two-way option contracts with suppliers. These contracts involve signing both call option contracts and put option contracts simultaneously for a comprehensive management of both the stock shortage risk and the shelf-life risk. The theoretical framework for supplier selection of two-way option contracts is presented in Figure 6.

If the quantity of emergency medical supplies in the government’s emergency reserve is insufficient, the government can exercise the call option and procure emergency medical supplies from the supplier. In cases where there is no demand for emergency medical supplies resulting from sudden disasters or when the demand is minimal, and there are remaining emergency medical supplies in the government’s emergency reserve pool, the government can request the supplier to repurchase the surplus emergency medical supplies, at the strike price specified in the put option contract.

By implementing a two-way option contract, both the stock shortage risk and the shelf-life risk can be effectively managed. The government incurs a certain option cost when entering into option contracts with suppliers, ensuring a balanced approach to address these risks without excessive costs. This framework allows for an effective response to stock shortage risks while mitigating the shelf-life risk faced by the government.

**Figure 7:**
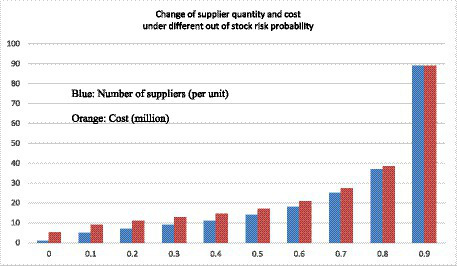
Chart of supplier selection quantity and cost change under different risk probabilities under two-way options.

**Figure 8:**
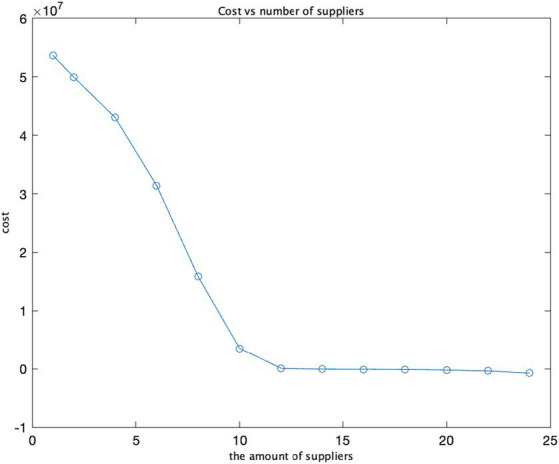
Cost and supplier quantity.

**Figure 9:**
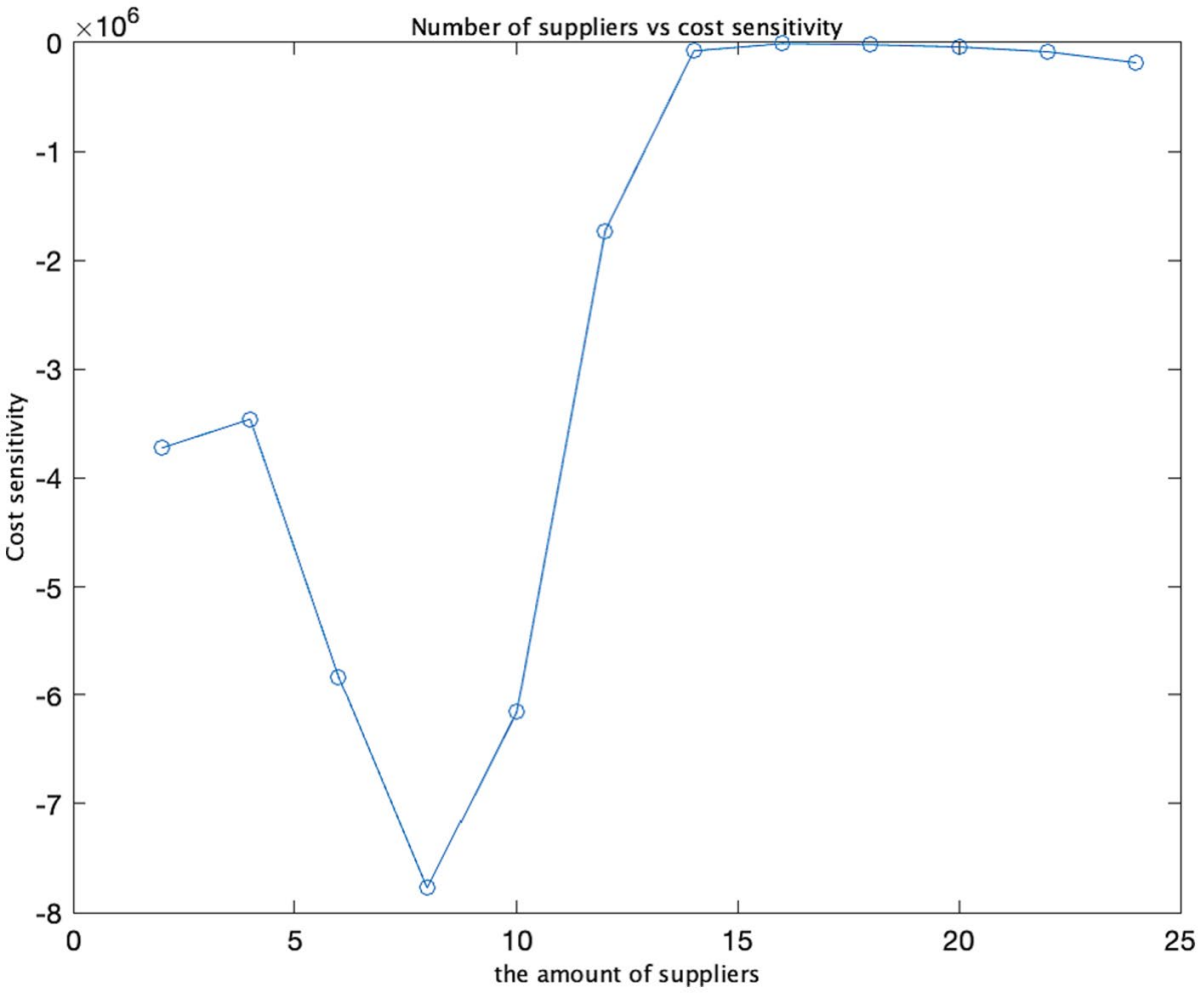
Supplier quantity and cost sensitivity.

**Figure 10:**
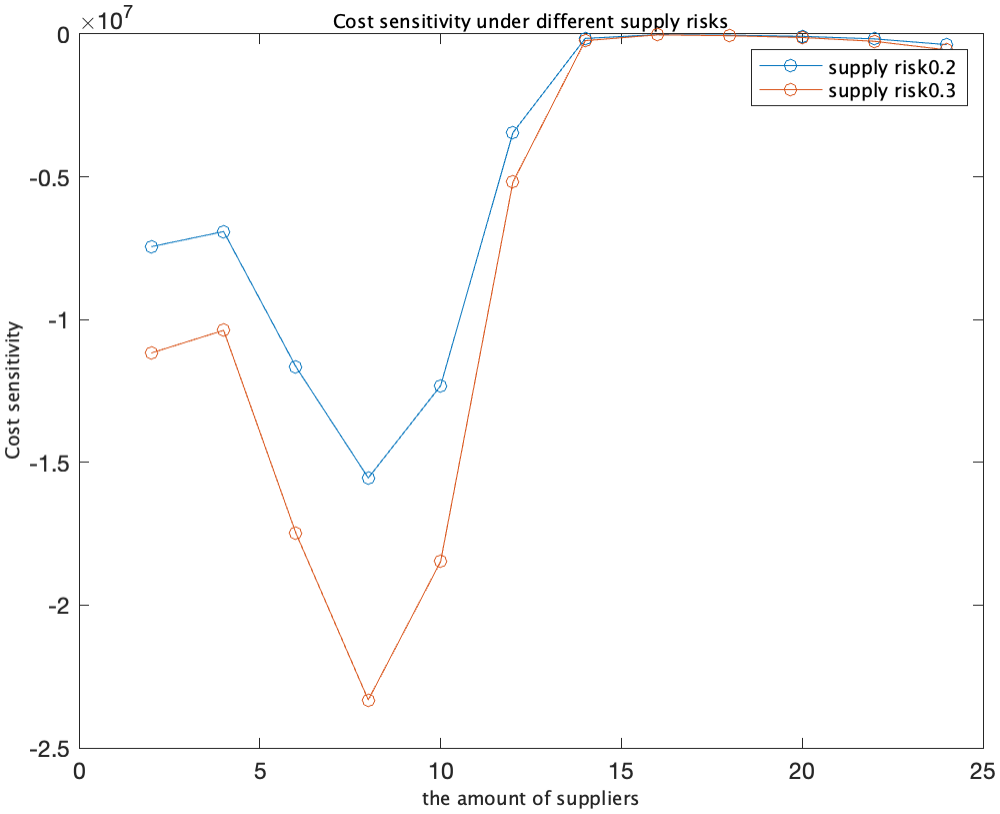
Cost sensitivity at different supply risks.

*PQ* is procurement cost of the government’s option reserve.

The two-way option model is formulated as follows: Equations 3.1–3.7.


(3.1) $$TC\left(n\right)\;=\;PQ_{u}+U_{n}+V_{u}+W_{u}-V_{d}$$

(3.2) $$Uu=a+bn^{\theta }+c/n+o(q_{u}+q_{d})n$$

(3.3) $$V_{u}=\overset{n}{\underset{i=0}{\sum }}C_{n}^{i}p^{n-i}{\left(1-p\right)}^{i}\overset{U}{\underset{Q_{u}+q_{u}i}{\int }}r\left[\xi -\left(Q_{u}+q_{u}i\right)\right]f\left(\xi \right)d_{\xi }$$

(3.4) $$W_{u}=\overset{n}{\underset{i=0}{\sum }}C_{n}^{i}p^{n-i}{\left(1-p\right)}^{i}\left\{\overset{U}{\underset{Q_{u}+q_{u}i}{\int }}\omega_{u}q_{u}if\left(\xi \right)d_{\xi }+\overset{Q_{u}+q_{u}i}{\underset{Q_{u}}{\int }}\omega_{u}\left(\xi -Q_{u}\right)f\left(\xi \right)d_{\xi }\right\}$$

(3.5) $$\begin{array}{c} V_{d}=\overset{n}{\underset{i=0}{\sum }}C_{n}^{i}p^{n-i}{\left(1-p\right)}^{i}\;\\ \left\{\begin{array}{c} \overset{Q_{d}-q_{d}i}{\underset{0}{\int }}\left[\left(Q_{d}-q_{d}i-\xi \right)\left(P-v\right)+\omega_{d}q_{d}i\right]f\left(\xi \right)d_{\xi }\\ +\overset{Q_{d}}{\underset{Q_{d}-q_{d}i}{\int }}\left(Q_{d}-\xi \right)\omega_{d}f\left(\xi \right)d_{\xi } \end{array}\right\} \end{array}$$

(3.6) $$\begin{array}{c} TC\left(n\right)=PQ+U_{n}+V_{u}+W_{u}-V_{d}=PQ+a\\ +bn^{\theta }+c/n+o\left(q_{u}+q_{d}\right)n+\overset{n}{\underset{i=0}{\sum }}C_{n}^{i}p^{n-i}\\ {\left(1-p\right)}^{i}\left\{\begin{array}{l} \overset{U}{\underset{Q_{u}+q_{u}i}{\int }}r\left[\xi -\left(Q_{u}+q_{u}i\right)\right]f\left(\xi \right)d_{\xi }+\overset{U}{\underset{Q_{u}+q_{u}i}{\int }}\omega_{u}q_{u}if\left(\xi \right)d_{\xi }\\ +\overset{Q_{u}+q_{u}i}{\underset{Q_{u}}{\int }}\omega_{u}\left(\xi -Q_{u}\right)f\left(\xi \right)d_{\xi }-\\ \overset{Q_{d}-q_{d}i}{\underset{0}{\int }}\left[\left(Q_{d}-q_{d}i-\xi \right)\left(P-v\right)+\omega_{d}q_{d}i\right]\\ f\left(\xi \right)d_{\xi }-\overset{Q_{d}}{\underset{Q_{d}-q_{d}i}{\int }}\left(Q_{d}-\xi \right)\omega_{d}f\left(\xi \right)d_{\xi } \end{array}\right\} \end{array}$$

(3.7) $$s.t.=\{\begin{array}{c} n\geq 1\\ n\in Z \end{array}$$

## 4 Results

Emergency medical supplies with high responsiveness and extended shelf life risk serve as case studies to examine the optimal number of suppliers within the context of two-way option contracts. Taking emergency medical supplies from Emergency medical suppliers in the Z region as an example, the government’s initial reserve under the two-way option contract is set at 3,000 boxes, the expected market emergency purchase price is 30 yuan per box. The strike price under the call option is 20 yuan per box, while the strike price under the put option is 10 yuan per box. The unit option cost for the two-way option stands at 10 yuan per box. Historical data indicates a maximum demand of approximately 6,000 boxes, with demand conforming to a normal distribution characterized by a mean value of 100 and a variance of 4, represented as ξ ~ *N* (100, 4). The government’s initial reserve purchase price is 15 yuan per box, and the government’s self-handled medical loss value is 10 yuan per box. The government purchases 800 boxes through call options and 600 boxes through put options from a single emergency provider under the two-way option. Based on recent data on supplier defaults after disasters in the Z region, the probability of supplier default risk ranges from 0.1 to 0.3. In this context, the following parameters are defined: *U* = 6,000, *Q* = 3,000, q_u = 800, q_d = 600, r = 30, ω_u = 20, ω_d = 10, o = 10, and *p* = 15. Specific model parameters include *a* = 5, *b* = 2, *θ* = 2, and *c* = 1. These parameters are incorporated into the optimal supplier quantity decision model, which considers both out-of-stock risk and shelf life risk. MATLAB software will be employed to simulate each parameter’s impact on the optimal supplier quantity within the model as they undergo individual changes (Table 4).

**Table 4:** Different out-of-stock risks affect the results of supplier quantity selection.

Out of stock risk probability	The amount of suppliers	Cost	Final number	Final cost
0.1	4.9423	88,332	5	89,363
0.2	6.6151	103,340	7	109,353
0.3	8.4465	119,820	9	127,672

As the supply risk escalates, the government’s optimal supplier count also rises gradually. When the supply risk is at 0.1, the optimal supplier count stands at 5. With a subsequent increase in supply risk to 0.2, the optimal supplier count elevates to 7. Further amplification of the supply risk to 0.3 necessitates an additional increase in the optimal supplier count to 9. Corresponding to the surge in supply risk, there is a discernible uptrend in final costs. When the supply risk escalates from 0.1 to 0.2, the final cost escalates from 89,363 to 109,353. Subsequently, with a further supply risk increase to 0.3, the final cost experiences another upswing, reaching 127,672 (Table 4). This underscores that, under circumstances of heightened supply risk, the government must incur a higher cost to ensure the availability of emergency medical supplies.

We have utilized three input variables: the number of suppliers, costs, and opportunity costs. By varying the opportunity cost values from 10 to 30, we calculated the ratio between the change in cost and the change in opportunity cost, known as cost sensitivity. As the opportunity cost escalates from 10 to 20 and 30, the cost increases by approximately 20,000 and 29,000, respectively. This suggests that the opportunity cost exerts a substantial influence on the total cost, and cost sensitivity amplifies as the opportunity cost rises. In the graph illustrating the relationship between opportunity cost and cost, costs surge as opportunity costs increase, aligning with the observed trend in cost sensitivity. In the graph representing the connection between opportunity cost and cost sensitivity, an analogous pattern emerges: as opportunity costs mount, cost sensitivity also intensifies, signifying a growing impact of opportunity cost on total costs.

The findings and sensitivity analysis of the model demonstrate its efficacy. The strategic determination of an optimal number of suppliers is imperative for safeguarding the security of the supply chain while concurrently minimizing costs (Figures 7–10).

## 5 Discussion and conclusions

### 5.1 Discussion

This research paper presents an analysis of the supplier selection process within the context of call option, put option, and two-way option contracts. It investigates the impact of various factors, such as opportunism cost, cooperation degree, and default risk, on the determination of the optimal number of suppliers. The following related areas warrant further exploration and improvement:

1. When the government engages in medical procurement, situations may arise that require emergency sourcing from the market, necessitating the selection of suppliers at different stages, such as early reserves and during periods of short supply. Therefore, it would be valuable to investigate the joint consideration of supplier selection across these three stages.
2. It is plausible for multiple disaster events to transpire during a single reserve period. It is crucial to further examine the concept of multi-cycle cooperation within the context of joint reserve operations between the government and suppliers.

### 5.2 Conclusion

The number of emergency medical suppliers chosen by the government plays a critical role in ensuring an effective supply of emergency medical supplies and addressing the breakdown of the emergency medical supply chain. This paper focuses on analyzing the selection of the number of suppliers in the field of emergency response. The government should consider factors such as responsivity, supply risk, procurement reserve cost, and the probability of disaster occurrence when selecting emergency medical suppliers. The risks of stock shortages and shelf-life are two external manifestations influenced by various scenario factors. To ensure the supply of emergency medical supplies and prevent the waste of expired emergency medical supplies, the government can effectively mitigate these risks by signing call option contracts and put option contracts with suppliers. From the perspective of minimizing the total procurement cost, this paper discusses the optimal quantity selection of emergency medical suppliers for the government under the risks of stock shortages and shelf-life.

1. The issue of option contract signing for emergency medical supplies with different attributes. Call option contracts are found to effectively mitigate supply risks for emergency medical supplies with high demand and long shelf-life. Put option contracts, on the other hand, help address the shelf-life risk associated with emergency medical supplies that have a short shelf-life. By applying two-way option contracts, which combine both call and put options, the government can make more informed decisions when procuring emergency medical supplies. This approach enhances the government’s ability to support emergency medical supplies while reducing the risk of shelf-life expiration and minimizing waste of resources.
2. The analysis of supply risk problem under the influence of multiple scenario factors. Emergency medical supplies with high responsivity are physically stored in the government reserve pool in the early stages. However, during large-scale emergency disasters, the limited space and budget of the government reserve pool may result in inadequate emergency medical supplies compared to the disaster’s needs, leading to the risk of stock shortages. On the other hand, when disasters do not occur or the scale is small, the demand for emergency medical supplies is low, and the emergency medical supplies stored by the government to respond effectively to sudden disasters may face the risk of shelf-life expiration.
3. The paper constructs an optimal supplier quantity selection model under option contracts. By considering the minimization of the total procurement cost, the study examines the selection of the number of government emergency medical suppliers while taking into account risks associated with the supply chain. The total purchase cost encompasses components such as option transaction costs, stock losses, emergency purchase costs, and shelf-life surplus value income. The paper introduces call option contract theory to address the risk of stock shortages and put option contract theory to mitigate the risk of shelf-life expiration. Furthermore, when both risks coexist, the concept of two-way option contracts is introduced to establish an optimal quantity selection model for suppliers under different option contracts. The proposed model for optimal supplier quantity decision provides a new perspective and scientific foundation for the government to address the challenge of supplier quantity selection in the presence of supply risks.

## Data availability statement

The original contributions presented in the study are included in the article/supplementary material, further inquiries can be directed to the corresponding author.

## Author contributions

HH: Conceptualization, Funding acquisition, Investigation, Supervision, Writing – review & editing. KZ: Validation, Visualization, Writing – review & editing, Software, Project administration, Formal analysis. XZ: Data curation, Methodology, Software, Writing – original draft.
